# A distant relationship?—investigation of correlations between DNA isolated from backspatter traces recovered from firearms, wound profile characteristics, and shooting distance

**DOI:** 10.1007/s00414-020-02374-1

**Published:** 2020-07-20

**Authors:** Jan Euteneuer, Annica Gosch, Philipp Cachée, Cornelius Courts

**Affiliations:** 1 Institute of Forensic Medicine, University Medical Center Schleswig-Holstein, Kiel, Germany; 2 Sachverständigenkanzlei Cachée, Pistoriusstrasse 6a, 13086 Berlin, Germany

**Keywords:** Molecular ballistics, Backspatter, Ballistic model, Shooting distance, Firing distance, Wound channel

## Abstract

In molecular ballistics, where traces originating from the use of firearms against biological targets are investigated, “backspatter” traces are of particular importance. This biological material comprising blood and tissue from the victim is propelled back from the bullet entry site towards the direction of the shooter and can consolidate and persist on the inner and outer surfaces of the firearm, from where it can be collected and analyzed. Thus, a connection between the weapon and the victim can be established solely by molecular biological trace analysis. For the criminalistic investigation of gun-related crimes, the determination of the distance between the weapon and the victim can be of critical importance in reconstructing the circumstances of a crime. In this study, we investigated possible correlations between the shooting distance and the amount of backspatter in/on the used firearm. To this purpose, we employed a previously established skull model and performed shootings in triplicates from various distances up to 50 cm with two types of handguns (pistol and revolver). Backspatter was collected from various sampling locations, and DNA contents were quantified. A post-shooting wound channel evaluation was conducted by optical and radiological evaluation. The obtained DNA yields varied considerably between replicates from the same and from different distances. In contrast, apart from contact shots, no meaningful differences were observable in wound channel evaluations. In summary, no meaningful correlation between backspatter distribution and DNA yields, the shooting distance and the condition of the wound channel could be established.

**Electronic supplementary material:**

The online version of this article (10.1007/s00414-020-02374-1) contains supplementary material, which is available to authorized users.

## Introduction

In forensic criminalistic casework, it is essential to not only detect but also interpret the traces and trace patterns at a crime scene, which includes trace material on the body of persons or on objects that are or might be connected to the circumstances of that crime. Biological traces are of major interest, especially in serious offenses against life or physical integrity, as they can hold clues not only for the identification of involved persons but also about the activities that led to their deposition [1]. In many world regions, firearm ownership is prevalent, gunshot related injuries are common, and consequently firearms are frequently employed in those offenses [2], thus a careful investigation of these objects in forensic casework is of integral relevance. Therefore, the term “molecular ballistics” had been introduced as moniker for a transdisciplinary approach with the aim of inferring information about the involved parties and the course of events of a crime involving firearm injury and death, based on the molecular analysis of the complex patterns of biological traces generated by shots at biological targets. An important fraction of these traces originate from a biophysical effect termed “backspatter” [3], in which a biological material (blood, tissue, etc.) is propelled out of a bullet’s entry site in the target back towards the direction of the firearm and shooter [4]. This long-known phenomenon [5] demonstrably generates persistent traces, which bear the potential to not only connect victims of firearm injuries with the used weapon in homicides [6] and suicides [7] but also support crime scene trace contextualization, e.g., by target zone identification by forensic RNA analysis [8] or for simultaneous investigation of nuclear DNA, mtDNA, mRNA, and miRNA [9].

A critical aspect in the investigation of gunshot incidents is the distance whence the shot was fired. This can be crucial, especially for short distances, e.g., for the verification or falsification of witness statements, the differentiation between accidentally or intentionally and even self-inflicted wounds, or for the distinction between suicide and homicide. The distances between muzzle and target are generally classified as (tight or loose) contact shots, near-contact shots, intermediate shots, and distant shots, with more or less smooth transitions between the categories and always depending on the type of firearm and ammunition [10, 11]. The application of molecular biological analysis of backspatter traces to estimate the shooting distance and/or discriminate between distance categories would provide an additional tool to support the objective, evidence-based reconstruction of the events that took place at a crime scene. These analyses include quantitative evaluation of nucleic acids in backspatter traces recovered from outer and inner surfaces of the firearm. While a qualitative approach had already been introduced by MacDonell and Brooks in 1977 [12], only current quantification techniques can achieve this purpose with required sensitivity. Grabmüller et al., in a pilot study from 2016 [13], were the first to relate nucleic acid quantification from backspatter traces with shooting distance. They detected traces of backspatter inside of firearms with sufficient amounts of nucleic acids to enable successful analysis of samples from up to 15- (DNA) and 30-cm (RNA) shooting distances.

In the present study, we aim to assess whether after replicated shots with different types of firearms at a realistic ballistic model of the human skull [14] a correlation of distribution of and DNA content from backspatter traces and/or the wound profile inferred from the gelatin core of the skull model with the shooting distance can be established.

## Materials and methods

### Blood collection and sample mixtures

Venous blood used for the generation of the sample mixtures was drawn by using venipuncture and collected in sterile K3 EDTA S-Monovettes® (Sarstedt, Germany) and was donated by two informed and consenting volunteers. Both donors neither had had any contact with the weapons or ammunition before or during the experimental shooting nor participated in the sample mixture and model preparation, the sample collection at the shooting site, or downstream processing. The employed sample mixtures were prepared for each donor separately and were a modification of the contrast mixture described elsewhere [15] without acrylic paint, hence termed “double-contrast” mixture. Each mixture was freshly prepared prior to the shooting events before assembling the ballistic models and consisted of the donated EDTA blood and Micropaque® (Guerbet, Germany) or Barilux® CT contrast agent (Sanochemia Diagnostics, Germany) in a ratio of 1:1.

### Preparation of the ballistic model

We employed and established an anatomically realistic ballistic skull model [14] which was used for every shooting session with a few deviations. Briefly, commercially available and anatomically correct hollow SYNBONE® skull models with rubber coating (model number 8880.G, SYNOBONE AG, Switzerland) were cleaned from the inside and doped with a tissue simulating spongy matrix soaked with the “double-contrast” mix and sealed in evacuated vacuum bags. The bags were placed directly behind the occipital bone as it is the most typical target location for homicidal gunshot injuries to the head [16]. The skulls were filled with type III ballistic gelatin (Honeywell Fluka™, Germany) prepared at a 10% concentration following Fackler’s instruction [17] and stored for about 36 h at 4 °C prior to each shooting event.

### Experimental shooting setup and firearms

The experimental shootings were conducted at the designated shooting area on the premises of the State Office of Criminal Investigation of Schleswig-Holstein (LKA-SH) in Kiel. The prepared skull models were fixed at the zygomatic process of the temporal bone with cork-lined lab clamps attached to a metal rod on a wooden board, which was oriented in such a way that the trajectory was leading towards the bullet trap (Supp Fig. 1). All shots were executed one-handedly by a trained professional without stabilization of the weapon to provide for realistic shooting conditions. To avoid contamination by the shooter, a sterile surgical gown (Lohmann & Rauscher, Germany), standard earloop facemasks (3M Health Care, Germany), and Micro-Touch® nitrile examination gloves (Ansell, Belgium) were worn while and changed before each shooting.

To represent firearms that are frequently encountered in routine casework, two common handguns, a pistol (Glock 19, 9-mm Luger) and a revolver (Smith & Wesson CTG, .38 Special) were employed in our study (Table 1). Each firearm was assigned to one of the blood donors, i.e., in all shots performed with the pistol, the skull models containing a “double-contrast” mix with blood from donor one were used, and in case of the revolver a “double-contrast” mix with blood from donor two were used, respectively. Shootings were conducted in triplicates per distance (0 cm, 5 cm, 10 cm, 15 cm, 20 cm, 30 cm, 50 cm) and weapon, and were aimed orthogonally for the occipital bone, minding the deviations caused by free-handed shots. After each shot, the skull models were carefully transferred into plastic bags for further evaluation.

**Table 1:** Weapon types, firearms, and ammunition

Type	Firearm	Manufacturer	Ammunition	Muzzle energy	Manufacturer
Pistol	Glock 19	Glock (Austria)	9-mm Luger FMJ	518 J	Sellier & Bellot (Czech Republic)
Revolver	CGT Model 36	Smith & Wesson (USA)	.38 Special NonTox TFMJ	363 J	Sellier & Bellot (Czech Republic)

*FMJ*, full metal jacket; *TFMJ*, total full metal jacket

Muzzle energy was obtained from the manufacturer

### Weapon sampling and cleaning

After each firing, the outer and inner surfaces of the respective weapon were sampled at 3 (pistol) and 2 (revolver) locations, respectively: sampling location A—the outside surface of the frame and slide, B—the inside surface of the barrel, and C—the outer surface of the barrel and area around the recoil spring (only pistol, with open slide). Sampling was conducted using DNA-free forensic nylon swabs (4N6 FLOQ Swabs Genetics, Copan Flock Technologies, Italy), applying the double-swab technique [18] with 100-μL HPLC gradient-grade water (Th. Geyer GmbH & Co. KG, Germany) for the wet swab. Remaining or additional visible backspatter traces were collected with a modified double-swab technique by employing a single swab with one-half dry and one-half moistened with 20-μL HPLC gradient-grade water.

Before using the firearms again, all surfaces were cleaned mechanically and chemically with Roti®-Nucleic Acid-free (Carl Roth GmbH, Germany) and distilled water. The barrel was scrubbed with soaked cotton pads (Vereinigte Filzfabriken AG, Germany), and the recoil spring was incubated in Roti®-Nucleic Acid-free for several minutes and thoroughly cleaned with cotton swabs. All parts were subsequently blow-dried with compressed air. After each cleaning, negative controls were taken from all sampling locations.

### DNA extraction, quantification, and STR profiling

DNA was extracted from the collected samples using the PrepFiler® Forensic DNA Extraction Kit (Thermo Fisher Scientific, USA) according to the manufacturer’s instructions, resulting in a final elution volume of 50 μL. The wet and dry swab pairs resulting from double-swab sampling were sequentially lysed in the same tube and lysis buffer, producing a combined eluate. If a swab was saturated with trace material, no sequential lysis was performed, but each swab of a pair was treated solely. From both “double-contrast” mixtures by each donor, three swabs with a defined volume of 20 μL were created at each shooting event and processed accordingly for normalization, to account for the physiological intraindividual variation of the white blood cell count. The mean of the triplicates from the first shooting event was taken as a default, and the values from the following events were used for correcting the obtained DNA yields of the backspatter samples.

The DNA concentration was determined by quantitative PCR (qPCR) using the PowerQuant® system (Promega, USA) on an Applied Biosystems™ 7500 fast Realtime PCR System (Thermo Fisher Scientific, USA). Quantification was performed in duplicates following the manufacturer’s instructions with a 2-μL DNA sample in a reduced reaction volume of 10 μL. This variant approach had been thoroughly validated for routine analysis in our laboratory. PCR inhibition (common at samples from weapon parts, especially from inside the barrel) as indicated by the PowerQuant® data analysis tool was noted, but disregarded for the evaluation of the autosomal DNA concentrations, as an effect on the quantification is only visible at high concentrations [19].

All concentrations were multiplied by the elution volume to obtain DNA amounts in ng. For each firearm, the values of every sampling location were also added up to a total amount of obtained DNA from collected backspatter. All values were compared, clustered, and evaluated for potential patterns and limits.

For one sample from each backspatter event and for all negative samples exhibiting an amount of quantifiable autosomal DNA above our internally validated analytical threshold of 0.4 pg/μL, short tandem repeats (STR) multiplex PCR was performed using the PowerPlex® ESX 17 Fast Kit (Promega, USA) according to the manufacturer’s protocol on an Applied Biosystems™ GeneAmp PCR System 9700 (Thermo Fisher Scientific, USA) with an optimal input amount of 0.5-ng DNA. PCR products were separated and detected on an Applied Biosystems™ 3500 Genetic Analyzer (Thermo Fisher Scientific, USA), and data analysis was done with the GeneMapper ID-X software version 1.5 (Thermo Fisher Scientific).

### Radiological imaging

Post-shot CT imaging was performed 24 h after shooting by multislice CT (Ingenuity Core or IQon Spectral CT, Philips, Netherlands) with the routinely used native cranial CT protocol scan with a tube voltage of 120 kV and tube currents defined by the automatic exposure control between 170 and 280 mAs in a spiral or helical mode, to obtain optimal and most reliable results. Reconstruction was performed with a bone and smooth kernel and iterative model reconstruction (IMR) with spacing of 0.8 and 2.5 mm. A sharper kernel with 0.8-mm spacing was found to be a suitable approach for the delineation of the contrast medium versus air versus gelatin along the wound channel. The image post-processing (multiplanar reconstruction with overlapping slices to achieve about 10-mm slice thickness in the AVG mode, to match the manual cut thickness) was done with InVesalius v3.1.1 (CTI, Brazil).

### Skull model and wound channel evaluation

After the radiological imaging, the skull models were photographed, and any peculiarities were noted. Afterwards, they were processed as described by Euteneuer et al. [14]. The gelatin brain simulant was extracted and serially cut into about 1-cm-thick slices perpendicular to the wound channel. A scanner (MP C306, Ricoh, Canada) was used to create images of the slices with a resolution of 600 dpi. The images were evaluated with ImageJ 1.52d (NIH, USA) by applying the polygon method [20, 21], where the end of the tears radiating from the permanent wound cavity is connected to create a polygon which reflects the extent of the temporal wound cavity and thus the dissipated kinetic energy and damage from the projectile on the gelatin brain simulant. The polygon perimeter was determined for every slice, and the arithmetic mean was calculated of the three replicates for every slice at each weapon-distance pair, thus creating an impression of the average damage along the wound channel. The results for every distance were compared with each other and evaluated for possible correlations to the shooting distance or DNA yield. This procedure was applied for the radiological images as well, and the results from both approaches were compared.

## Results

### DNA quantification and STR profiling

The obtained quantification results in ng/μL were converted into total DNA yields. The yields of both swabs employed in the double-swab procedure were added up to a total DNA yield for each sampling location, and DNA yields from each sampling locations were summed up to obtain the total DNA yield per firearm and distance. Additional yields from traces collected from weapon parts not representing the sampling locations A, B, or C (i.e., the backside of the slide (pistol) and inner or outer parts of the cylinder, recoil shield, and trigger guard (revolver)) were also added to the DNA amount per firearm and distance. These total amounts are presented in Table 2 with a logarithmic scale to account for the wide distribution of values. For a complete view of all values at each distinct sampling location, see Supp. Table 1.

**Table 2:** Total DNA yields from the replicates of each firearm per distance

DNA [ng]	Pistol							Revolver						
	0 cm	5 cm	10 cm	15 cm	20 cm	30 cm	50 cm	0 cm	5 cm	10 cm	15 cm	20 cm	30 cm	50 cm
0					0	0	0			0			0	0
							0							0
0.01														0
									0.02					
							0.10					0.17		
0.1														
1		0.87	0.77			0.76			0.57	0.54	0.76			
			2.18	1.88	2.10	1.57			0.95	1.25				
10				24										
		43			58			80			58			
		99	89					96						
100	457			170								243	129	
	571							586			398		217	
1000	861											931		
Sum	1889	143	92.0	196	60.1	2.33	0.10	762	1.54	1.79	457	1174	348	0

Values are rounded

The DNA yields from subsequent shooting events were corrected with regard to the first shooting session to account for the physiologically fluctuating white blood cell (WBC) counts of the donors (correction factors between 1.09 and 2.80), except for those values ranging below the analytical threshold of 0.4 pg/μL for successful STR profiling, to avoid false positives. These corrections were performed for each donor independently; the WBC counts of both donors (and thus both weapons) were not adjusted to each other, as the firearms were treated as separate experiments.

### General appearance of backspatter and STR profiling results

In general, traces of backspatter could manifest on the outer or inner surfaces of both the respective firearms (examples seen in Supp. Fig. 2). DNA from backspatter was quantified and STR-profiled from 17 of 21 pistol and 16 of 21 revolver shots. Four pistol (at 20, 30, and 50 cm) and five revolver samples (10, 30, and 50 cm) had no detectable biological traces or yielded DNA amounts below the analytical threshold. Two samples, recovered from 5-cm (revolver) and 50-cm (pistol) distance shots, with a DNA concentration of 0.4 and 0.7 pg/μL failed to create a profile. For the remaining 33 samples, STR profiling resulted in a full profile of the donor in 26 cases. In three samples, only the AMEL locus had dropped out reproducibly, while otherwise, a flawless profile had emerged. Nine samples exhibited from one up to four small extra alleles (all but two below 10% average peak height, and the two remaining between 15 and 18% in stutter positions, respectively) and were thus considered as drop-in alleles negligible for the evaluation of the DNA amount attributable to the respective blood donor.

### Distribution of DNA yields per firearm type

**Fig. 1:**
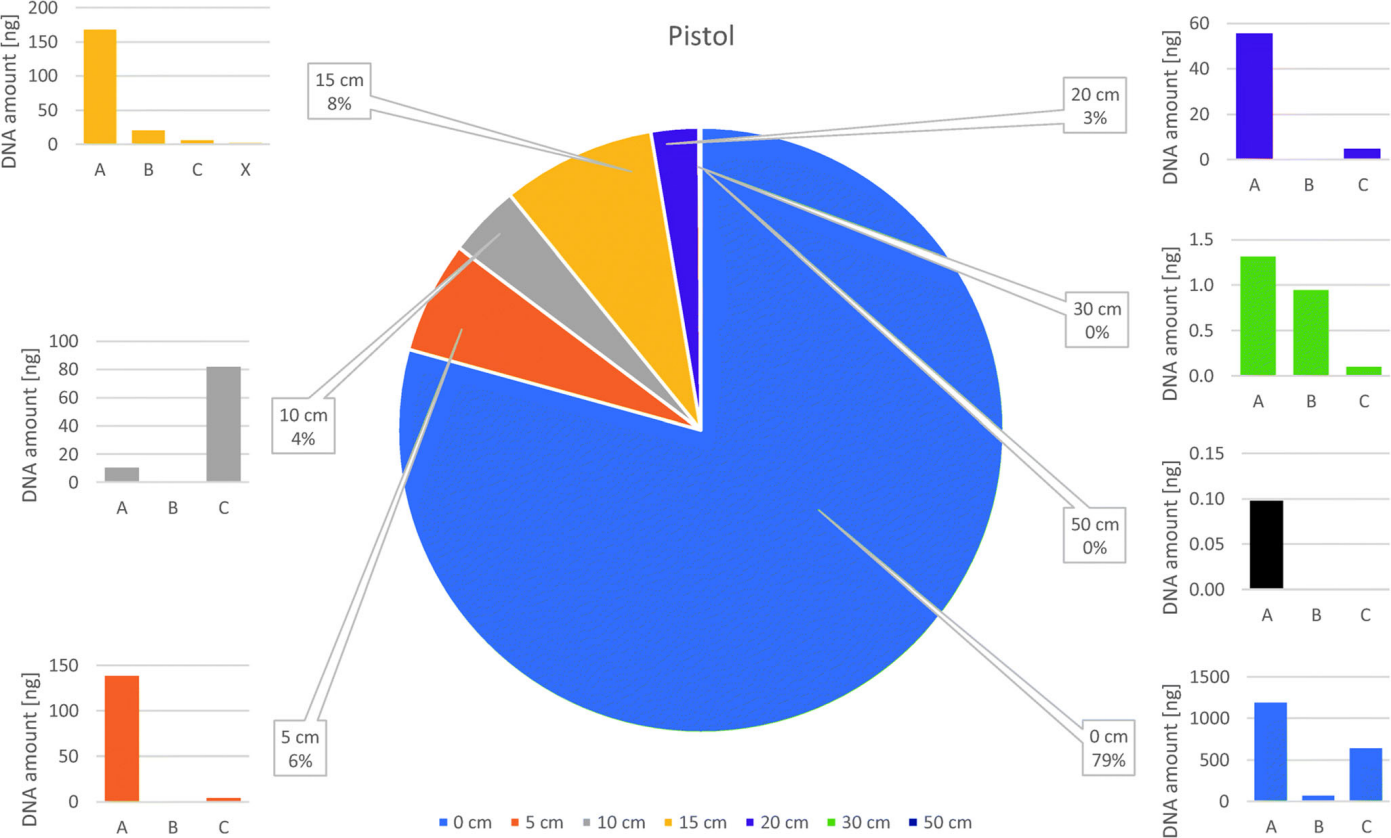
Distribution of DNA yields after pistol shots. Pie chart depicts the sums of the triplicates’ quantified DNA, extracted from all backspatter traces for the indicated shooting distances. Bar charts show the distribution of values at each sampling location as the sum of the triplicates. A Outside surface of the frame and slide. B Inside surface of the barrel. C Outer surface of the barrel and area around the recoil spring (open slide). X Other surfaces (backside of slide)

**Fig. 2:**
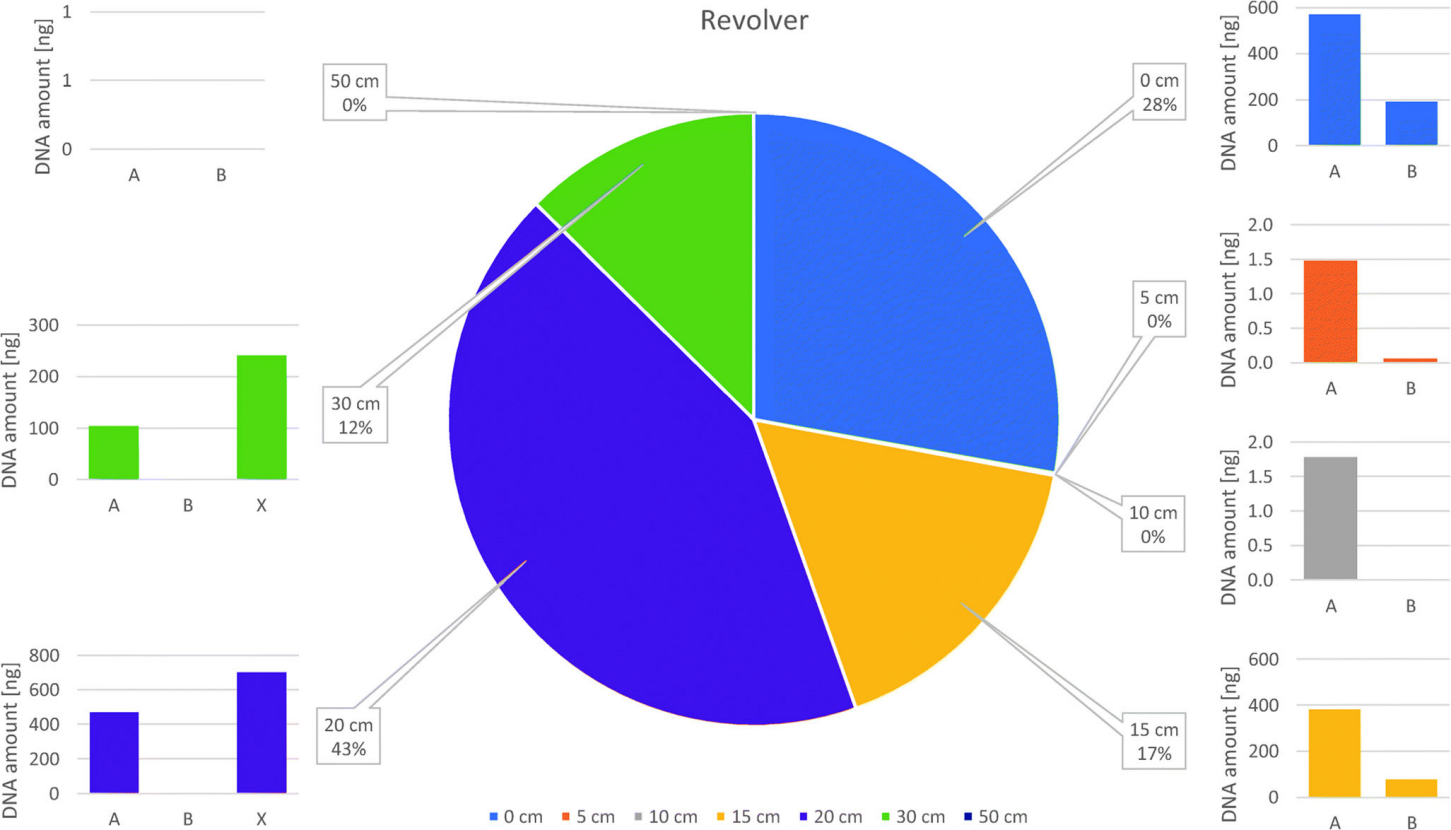
Distribution of DNA yields after revolver shots. Pie chart depicts the sums of the triplicates’ quantified DNA, extracted from all backspatter traces for the indicated shooting distances. Bar charts show the distribution of values at each sampling location as the sum of the triplicates. A Outside surface of the frame. B Inside surface of the barrel. X Other surfaces (cylinder, trigger guard, recoil shield)

Figures 1 and 2 depict the summarized DNA yields from all three replicates by the distance, and for each sampling location within the distances. In the case of the pistol, the highest amounts of total backspatter DNA were recovered after the three contact shot replicates (extensive backspatter seen in Supp. Fig. 2A). When placed in a descending order, the remaining values appear to be randomly distributed among the distances until the farthest distance of 50 cm, as shown in Table 2, yet with (already) decreasing amounts by the replicates from 30 cm. Hence, no correlation of DNA yield from backspatter and shooting distance could be established. There were even replicates produced at longer distances with higher DNA yield than replicates from closer distances, respectively.

The same was observed with the revolver shots, yet those exhibited distinctly lower values in the three replicates from 5- and 10-cm shooting distances (between 0 and 1.25 ng) as compared with the replicates from longer distances, which produced at least one or two values that were higher, by several orders of magnitude (between 0 and 930.6 ng), than those from the shorter distances. Notably and in contrast with the pistol, the highest DNA amount in a revolver sample was measured in a replicate from a 20-cm shooting distance. Consequently, the standard deviation of the replicate values per distance and firearm was of the same order of magnitude than the mean.

### Distribution of DNA yields per sampling location

With regard to the sampling location, both pistol and revolver produced quantifiable backspatter traces at 15 of 20 occasions for sampling location A. No backspatter was found on the outside surfaces of the pistol after one shot from a 20-cm distance and two shots from 50 cm, and after one shot from each 10- and 30-cm distance as well as after all three 50-cm shots for the revolver. In total, all traces from sampling location A add up to 1562.0-ng DNA for the pistol and 1530.0-ng for the revolver.

As expected, due to the small target zone, the inside of the barrel (sampling location B) exhibited the lowest proportion of samples in terms of amplifiable amounts of DNA (pistol: 10/21 and revolver: 8/21) and also total DNA yields (sum of all pistol results: 85.5 ng, revolver: 268.5 ng). Only contact shots produced quantifiable traces inside the barrel for all three replicates from both weapons (pistol sum: 64.1 ng and revolver: 191.2 ng). With the exception of one shot by each weapon from 15 cm and one revolver shot from 20 cm, no sample contained more than 1-ng DNA. No biological material was detected in replicates from 10- and 50-cm distances from the pistol, and from 10-, 30- and 50-cm distances from the revolver shots. In addition, samples from inside the barrel tended to exhibit signs of slightly increased PCR inhibition and DNA degradation in the PowerQuant analysis in comparison with replicates from sampling locations A and C (data not shown).

The pistol-exclusive sampling location C was determined to be a useful location for trace recovery as compared with sampling location B inside of the barrel. While most of the total DNA yield originated from the contact shot replicates (636.4 of 733.3 ng), quantifiable traces were also found in at least 2 of 3 replicates of the distant shots (in total 14/21), except for shots from a 50-cm distance, and demonstrated increased yields with a mean of 5.3 ng compared with 0.2 ng in samples from inside the barrel (excluding contact shots).

DNA yields from additional samples which, as mentioned above, had been recovered from other surfaces than sampling locations A, B, or C amounted to 1.9 ng for the pistol (from only one shot from a 15-cm distance) and 941.8 ng for the revolver, the latter originating from two shots from 20- and 30-cm distances, respectively.

### Negative controls and sample integrity

Quantifiable amounts of DNA with concentrations above the internal analytical threshold of 0.4 pg/μL were detected in 16 out of 114 negative samples (Supp. Table 2). The resulting values were put in relation to the DNA yield of the following shot at the corresponding sampling location. They were considered as negligible when either their ratio was below 5% of that DNA yield (13/16) or when their concentration was so low that no successful STR profiling had been obtained (2/16). In one remaining negative sample (pistol, 30-cm distance, sampling location C), a 7-fold higher DNA concentration was measured than in the corresponding next sample (0.6 pg/μL, just above the internal analytical threshold). This sample was disregarded for the total DNA sum of the respective pistol value in Table 2 and marked accordingly in Supp. Table 1.

As expected for DNA traces recovered from used firearms, some samples exhibited signs of degradation in the PowerQuant analysis system. This, however, had no observable effect on STR profiling.

### Examination of the wound cavity

The polygon method was applied for all wound channels in the same manner and also for cavities exhibiting shooting artifacts, like small “bone” fragments from the skull model, which were carried inside by the bullet. In one pistol contact shot, the plastic bag containing the “double-contrast” mixture had been dragged inside the wound channel, yet after examining and careful removal, it did not appear to have influenced the length or number of tears (all values are presented in Supp. Table 3).

**Fig. 3:**
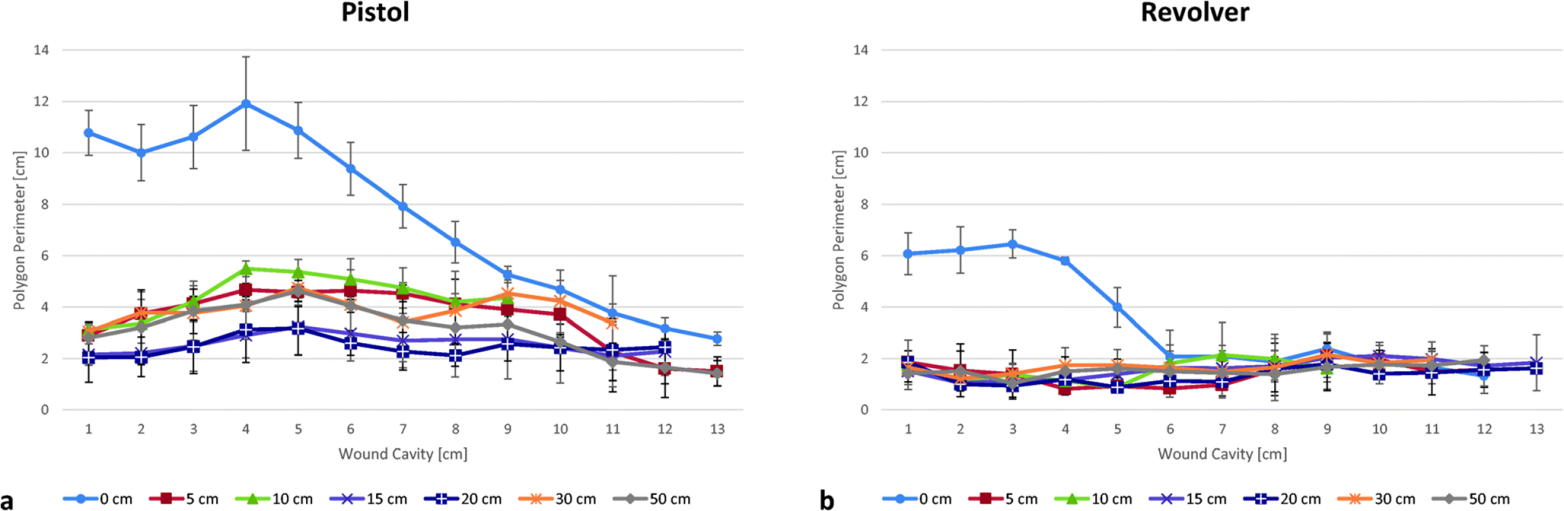
Combined polygon perimeter results by optical evaluation. Gelatin brain simulants were removed from the skull models 24 h after shooting and cut into 1-cm slices perpendicularly to the trajectory and scanned for optical evaluation using ImageJ v.1.52. Projected values are means of the three shots, with standard deviation. **a** Results after shots with Glock 19, 9-mm Luger. **b** Results after shots with Smith & Wesson CGT 36, .38 Special

In general, the gelatin slices allowed for a better evaluation and quantification of the tears than the radiological images, which at some point failed to visualize fine tears in cases where no “double-contrast” mixture was dragged far enough into the cavity and/or could diffuse through their whole length. In consequence, the radiologically determined values are comparable in their tendencies, but show slightly lower values than the manually prepared slices. The graph corresponding to the pistol and revolver wound channels is shown in Fig. 3 (radiological graphs are shown in Supp. Fig. 3). With both firearms, the wound profiles of contact shots are clearly distinguishable from those of distant shots, due to the vast destructive potential by the muzzle gasses [22].

Between the distant shots, however, no correlation between the amount of collected DNA and polygon perimeter values could be established. Replicates with higher DNA yields exhibited higher, lower, or similar wound channel values than replicates with lower DNA yields, regardless of distance. For instance, two replicates of the revolver shots from a 20-cm distance produced vastly different backspatter traces containing DNA amounts of 0.170 ng and 930.6 ng, respectively, while exhibiting very comparable values, with the polygon perimeters from all slices along the wound channel totaled up to 19.655 cm and 20.669 cm. For the pistol, higher values—as expected from ammunition with higher kinetic energy—and larger deviations between the shots were measured (between 1.4- and 5.5-cm polygon perimeter in a single slice) as compared with the revolver (0.8- to 2.9-cm perimeter); still, neither DNA amount nor shooting distance correlated in any meaningful way with the wound channel values.

## Discussion

In this study, we assessed possible relationships or proportional correlations between the presence and amount of backspatter retrieved from inner and outer surfaces of two types of firearms and increasing shooting distance. By delivering free-handed shots at fixed, previously established skull models [14], we aimed for a compromise between realistic and controlled shooting conditions. Yet, our data, resulting from two handguns and downstream processing-comprising techniques and equipment as used in standard forensic laboratories, exhibited considerable variation of DNA yields, within replicates from the same and between different distances, even taking into account our limited number of samples. Even DNA yields from close contacts were either outranged by more distant shots in case of the revolver or did not sufficiently outrange yields from more distant shots to infer a forensically relevant rule (pistol). An increased number of experimental shootings would allow for the application of more advanced statistical, e.g., probabilistic, approaches which could take into account the influence of the weapon and ammunition in use, persistence time, sampling technique, and DNA extraction and quantification methods. However, the large variability observed in our dataset strongly indicates that the inference of the shooting distance from amounts of backspatter recovered from different firearm surfaces will be associated with a large uncertainty, and it remains to be seen if such an approach could mend the risk of misinterpretation for isolated real case scenarios. While the handguns included in this study are very commonly encountered in and thus highly representative for forensic routine casework, further research should take into account additional types of firearms, as our findings may not be applicable to other weapons without experimental verification. Further studies can be based on our established model setup but may require additional modifications depending on the type of firearm tested. For instance, in a previous work, we demonstrated that shotgun blasts with slugs lead to total destruction of the model and hence prevent typical backspatter mechanisms [14].

Unsurprisingly, the largest amounts of DNA from backspatter traces were collected from sampling location A, i.e., from outer surfaces of the firearm, which are most exposed to and freely accessible for backspatter. In real cases of gun-related crimes though, especially in homicides, such traces might be removed by cleaning and/or environmental conditions, contaminated or otherwise interfered with, corroborating the argument of Courts et al. that trace recovery from used firearms must be extended to all available surfaces, hence including inner surfaces [6]. For the securing of evidence in real crime cases involving pistols, we therefore recommend to also investigate sampling location C, the outer surface of the barrel, and the area around the recoil spring with open slide, as we had previously observed that the recoil process with concurrent slide displacement is temporospatially correlated with the backspatter process [14]. Regarding those surfaces not directly exposed to the environment, we detected at sampling location C higher amounts of DNA (apart from shots fired from a 15-cm distance) with overall increased quality from all distances compared with the inside of the barrel (sampling location B).

Concerning the quantification, given that a “double-contrast” mix of EDTA blood and contrast agent in a ratio of 1:1 was used, it could be argued that the quantified amounts should be doubled to produce more realistic values. We decided against this, on the one hand to avoid bias and on the other hand arguing that counts of normal white blood cells per μL blood vary widely between individuals as well as depend on physiological conditions (normal WBC count of about 4.3–10 × 10^3^/μL [23]), and thirdly because of the pronounced differences between the obtained values, which require a more general evaluation in orders of magnitude, thus rendering a small systematic correction factor of no avail. In addition to that, the model setup cannot perfectly emulate a real human head with physiological tissues and a vascular system. Thus, while backspatter amounts can still be compared systematically, each actual value—even if being comparable to real cases of backspatter (e.g., for the barrel reported in [7])—needs to be treated with caution. Employing a ballistic model system imitating the human head with an anatomically correct bone simulant is nonetheless a very appropriate way to artificially emulate realistic head shot scenarios, as the energy distribution inside the skull as a “rigid case” is different and the pressure generated by the collapsing temporal cavity is higher as compared with soft tissue or simulants like gelatin or soap blocks without casing [24].

In general, our findings (albeit with the abovementioned limitations), resulting from a more systematic setup than in a previous study [13] by employing a realistic skull model, technical replicates, and a controlled environment, are still in agreement with observations, e.g., by MacDonell [12] and Stone [25], who evaluated 1200 suicide cases involving firearms for the mere presence of blood in the used weapons, or Karger [26], stating that no simple conclusions should be drawn by the mere presence (of a certain amount) or absence of backspatter regarding the distance of close and intermediate range shots.

The estimation of the firing distance is a complicated problem. As of today, the macroscopic and morphologic evaluation of the bullet wound and the surrounding skin and tissue for imprints, soot, powder tattooing, bruises, etc. in combination with a gunshot residue (GSR) analysis still appears to be the most productive approach for reliable differentiation between certain distances [11]. Particularly, the detection, quantification, and correlation of GSR with the shooting distance has been (with a focus on staining, e.g., [27]) and still is a promising field of research, including GSR detection via micro-CT [28] and the analysis of inorganic GSR [29] by emission spectrometry and mass spectroscopy, which, however, require advanced lab equipment. Hlavaty et al. performed a microscopic, histological analysis of the tissue surrounding the bullet entry site to estimate the shooting distance but also concluded that this approach is not suitable for forensic routine analysis [30].

Analysis of the wound channels resulted neither in a useful associability with the DNA quantification results nor usability to distinguish near, intermediate, or higher distances. In general, higher polygon perimeter values were found for the pistol shots as compared with the revolver. This was expected for ammunition with higher-energy (Table 1) shot at gelatin [31]. The muzzle gasses entering the wound cavity when contact shots are applied display severe wounding potential in real gunshot cases [10] and exhibited the same destructive potential in this study as was previously described by Euteneuer et al. [14] and elsewhere, e.g., as demonstrated by Schyma et al. [22, 32, 33]. While the polygon perimeters showed hardly any difference between the various shooting distances for revolver shots (despite containing bone splinters inside nearly every wound channel), the measured values by the pistol showed larger deviations between their replicates, with the 15- and 20-cm distances exhibiting slightly lower values than the rest. The deviations could in part be explained by small variations of the angle in which, and exact position where, the bullet penetrated the model, which cannot be controlled in free-handed shooting. More test firings are required to allow for a more in-depth assessment.

The wound channel evaluation by manual cutting of the gelatin brain simulant and scanning of the slices performed better in our hands than the CT image analysis. Arguable, this is due to the artificial structure of the model. In real cases, human brains affected by gunshot injury can rarely be processed in this manner; hence, CT imaging—even lacking contrast agent—is the means of choice for visualizing the wound channel [34]. The results of radiological analyses and imaging will improve by the involvement of experts in forensic radiology and wound ballistics as well as appropriate software for diagnostic purposes. However, this was not the main focus of this study.

## Conclusions

In our study, we did not find any meaningful correlation between amount and distribution of backspatter in and on firearms and the shooting distance. Neither the presence nor the absence of (a certain amount of) backspatter from a single shooting incident can be safely attributed to a possible distance below 50 cm. Based on our findings, we therefore advice against estimating the shooting distance from a quantitative backspatter trace analysis in case of gunshot injury, as this could lead to false assumptions.

Furthermore, wound channel evaluation only indicates close contact shots as seen as a consequence of the distinct damaging potential of the muzzle gasses. Wound profiles of shots from various distances are not distinguishable and do not correlate with a certain amount of backspatter.

As backspatter is a phenomenon that occurs very shortly after the projectile has entered the target, it temporally coincides with the recoil process of a pistol, which thus exposes the outer surface of the barrel and area around the recoil spring, rendering these surfaces relevant sources for the recovery of backspatter trace material.

## Electronic supplementary material

ESM 1 (XLSX 22 kb)

ESM 2 (XLSX 11 kb)

ESM 3 (XLSX 44 kb)

ESM 4 (PPTX 16854 kb)

## Data Availability

Raw and additional data are available upon request.

## References

[1] Gill P, Hicks T, Butler JM (2020). DNA commission of the international society for forensic genetics: assessing the value of forensic biological evidence-guidelines highlighting the importance of propositions. Part II: evaluation of biological traces considering activity level propositions. Forensic Sci Int Genet.

[2] Naghavi M, Marczak LB, Kutz M (2018). Global mortality from firearms, 1990-2016. JAMA.

[3] Stephens BG, Allen TB (1983). Back spatter of blood from gunshot wounds—observations and experimental simulation. J Forensic Sci.

[4] Courts C, Madea B, Schyma C (2012). Persistence of biological traces in gun barrels-an approach to an experimental model. Int J Legal Med.

[5] Brüning A, Wiethold F (1934). Die Untersuchung und Beurteilung von Selbstmörderschußwaffen. Dtsch Z Gesamte Gerichtl Med.

[6] Courts C, Gahr B, Madea B, Schyma C (2014). Persistence of biological traces at inside parts of a firearm from a case of multiple familial homicide. J Forensic Sci.

[7] Schyma C, Madea B, Courts C (2013). Persistence of biological traces in gun barrels after fatal contact shots. Forensic Sci Int Genet.

[8] Lux C, Schyma C, Madea B, Courts C (2014). Identification of gunshots to the head by detection of RNA in backspatter primarily expressed in brain tissue. Forensic Sci Int.

[9] Grabmüller M, Schyma C, Euteneuer J, Madea B, Courts C (2015). Simultaneous analysis of nuclear and mitochondrial DNA, mRNA and miRNA from backspatter from inside parts of firearms generated by shots at “triple contrast” doped ballistic models. Forensic Sci Med Pathol.

[10] Di Maio VJM (2016) Gunshot wounds. Practical aspects of firearms, ballistics, and forensic techniques, third edition. CRC Series in Practical Aspects of Criminal and Forensic Investigations. CRC Press, Boca Raton

[11] Knight B, Saukko PJ (2004) Knight’s forensic pathology, 3rd ed. Distributed in the United States of America by Oxford University Press, London, New York

[12] MacDonell HL, Brooks BA (1977) Detection and significance of blood in firearms. Leg Med Annu: 183–199 593036

[13] Grabmüller M, Cachée P, Madea B, Courts C (2016). How far does it get?-the effect of shooting distance and type of firearm on the simultaneous analysis of DNA and RNA from backspatter recovered from inside and outside surfaces of firearms. Forensic Sci Int.

[14] Euteneuer J, Gosch A, Cachée P, Courts C (2019). Evaluation of the backspatter generation and wound profiles of an anatomically correct skull model for molecular ballistics. Int J Legal Med.

[15] Schyma C, Lux C, Madea B, Courts C (2015). The ‘triple contrast’ method in experimental wound ballistics and backspatter analysis. Int J Legal Med.

[16] Karger B, Billeb E, Koops E, Brinkmann B (2002). Autopsy features relevant for discrimination between suicidal and homicidal gunshot injuries. Dtsch Z Gesamte Gerichtl Med.

[17] Fackler ML, Malinowski JA (1988). Ordnance gelatin for ballistic studies. Detrimental effect of excess heat used in gelatin preparation. Am J Forensic Med Pathol.

[18] Pang BCM, Cheung BKK (2007). Double swab technique for collecting touched evidence. Leg Med (Tokyo).

[19] Holmes AS, Houston R, Elwick K, Gangitano D, Hughes-Stamm S (2018). Evaluation of four commercial quantitative real-time PCR kits with inhibited and degraded samples. Int J Legal Med.

[20] Schyma CWA (2010). Colour contrast in ballistic gelatine. Forensic Sci Int.

[21] Schyma C, Madea B (2012). Evaluation of the temporary cavity in ordnance gelatine. Forensic Sci Int.

[22] Schyma C, Bauer K, Müller R, Brünig J, Gotsmy W (2020). The influence of muzzle gas on the temporary cavity. Int J Legal Med.

[23] Dörner K, Battista H-J (2006) Klinische Chemie und Hämatologie. 69 Tabellen, 6., aktualisierte Aufl. Taschenlehrbuch. Thieme, Stuttgart

[24] Madea B (2014). Handbook of forensic medicine.

[25] Stone IC (1992). Characteristics of firearms and gunshot wounds as markers of suicide. Am J Forensic Med Pathol.

[26] Tsokos M (ed) (2008) Forensic pathology reviews, Volume 5, 1. Aufl. Forensic Pathology Reviews. Humana Press, s.l.

[27] Zeichner A, Glattstein B (2002). Recent developments in the methods of estimating shooting distance. ScientificWorldJournal.

[28] Cecchetto G, Giraudo C, Amagliani A, Viel G, Fais P, Cavarzeran F, Feltrin G, Ferrara SD, Montisci M (2011). Estimation of the firing distance through micro-CT analysis of gunshot wounds. Int J Legal Med.

[29] Merli D, Amadasi A, Mazzarelli D et al (2018) Comparison of different swabs for sampling inorganic gunshot residue from gunshot wounds: applicability and reliability for the determination of firing distance. J Forensic Sci. 10.1111/1556-4029.13870 10.1111/1556-4029.13870 29975990

[30] Hlavaty L, Roquero L, Amley J, Root K, Ishikawa M, Koopmeiners A, Zhao L, Sung LM (2019). Discordance of gross and histologic findings in estimating the range of fire of gunshot wounds. J Forensic Sci.

[31] Zhang J, Yoganandan N, Pintar FA, Gennarelli TA (2005). Temporal cavity and pressure distribution in a brain simulant following ballistic penetration. J Neurotrauma.

[32] Schyma C, Müller R, Brenčičová E, Brünig J (2018). Distortion of the temporary cavity and its influence on staining in firearm barrels. Forensic Sci Med Pathol.

[33] Schyma C (2012). Wounding capacity of muzzle-gas pressure. Int J Legal Med.

[34] Stevenson T, Carr DJ, Harrison K, Critchley R, Gibb IE, Stapley SA (2020). Ballistic research techniques: visualizing gunshot wounding patterns. Int J Legal Med.

