# Supplementary material for: A distant relationship?—investigation of correlations between DNA isolated from backspatter traces recovered from firearms, wound profile characteristics, and shooting distance
Source: Int J Legal Med. 2020 Jul 20;134(5):1619–28. doi: 10.1007/s00414-020-02374-1 (PMC8211572; doi:10.1007/s00414-020-02374-1)
Supplement: Supplementary file 4 — (PPTX 16854 kb) [file 414_2020_2374_MOESM4_ESM.pptx]

## Slide 1
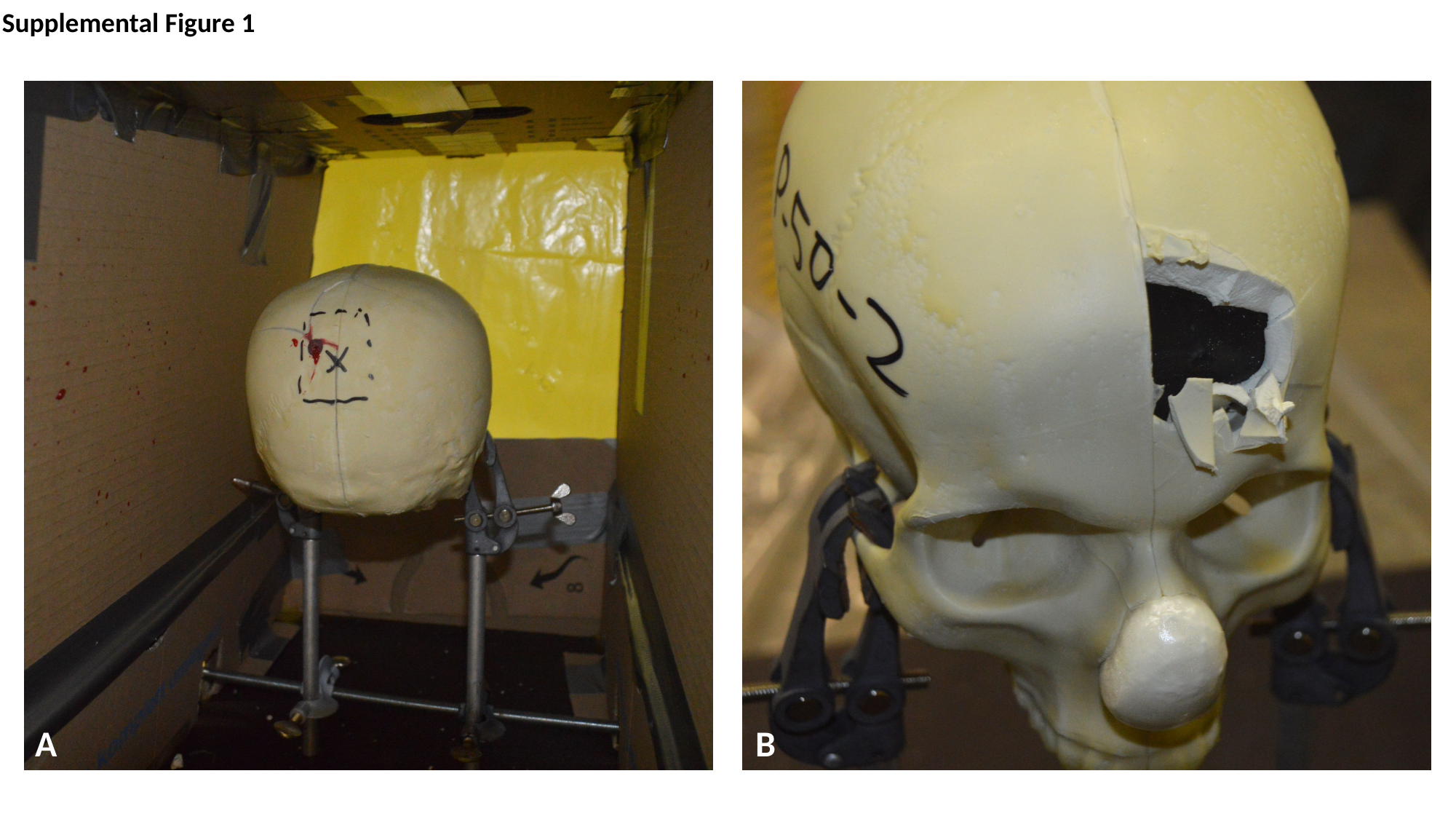

Supplemental Figure 1
A
B

## Slide 2
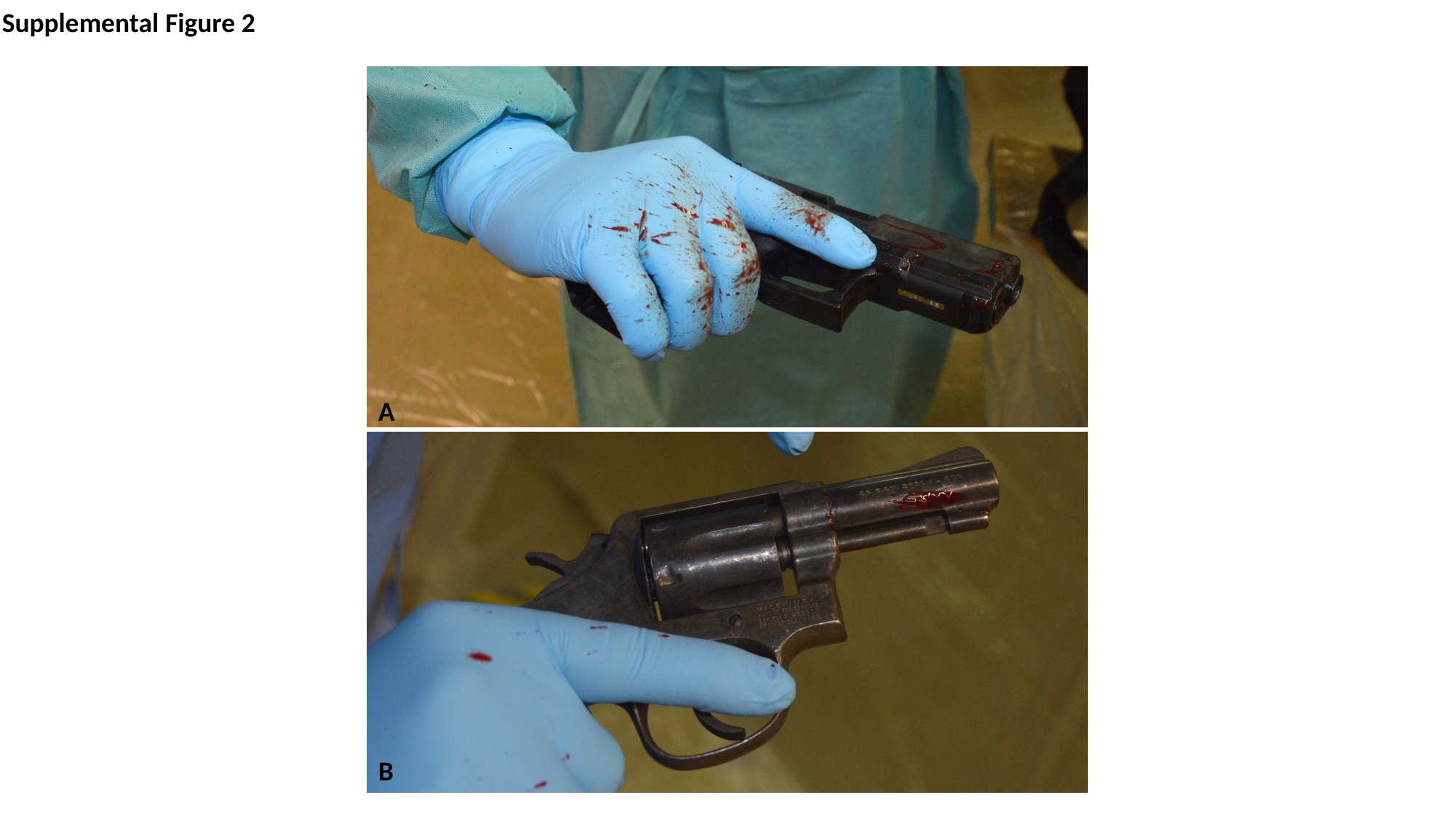

Supplemental Figure 2
A
B

## Slide 3
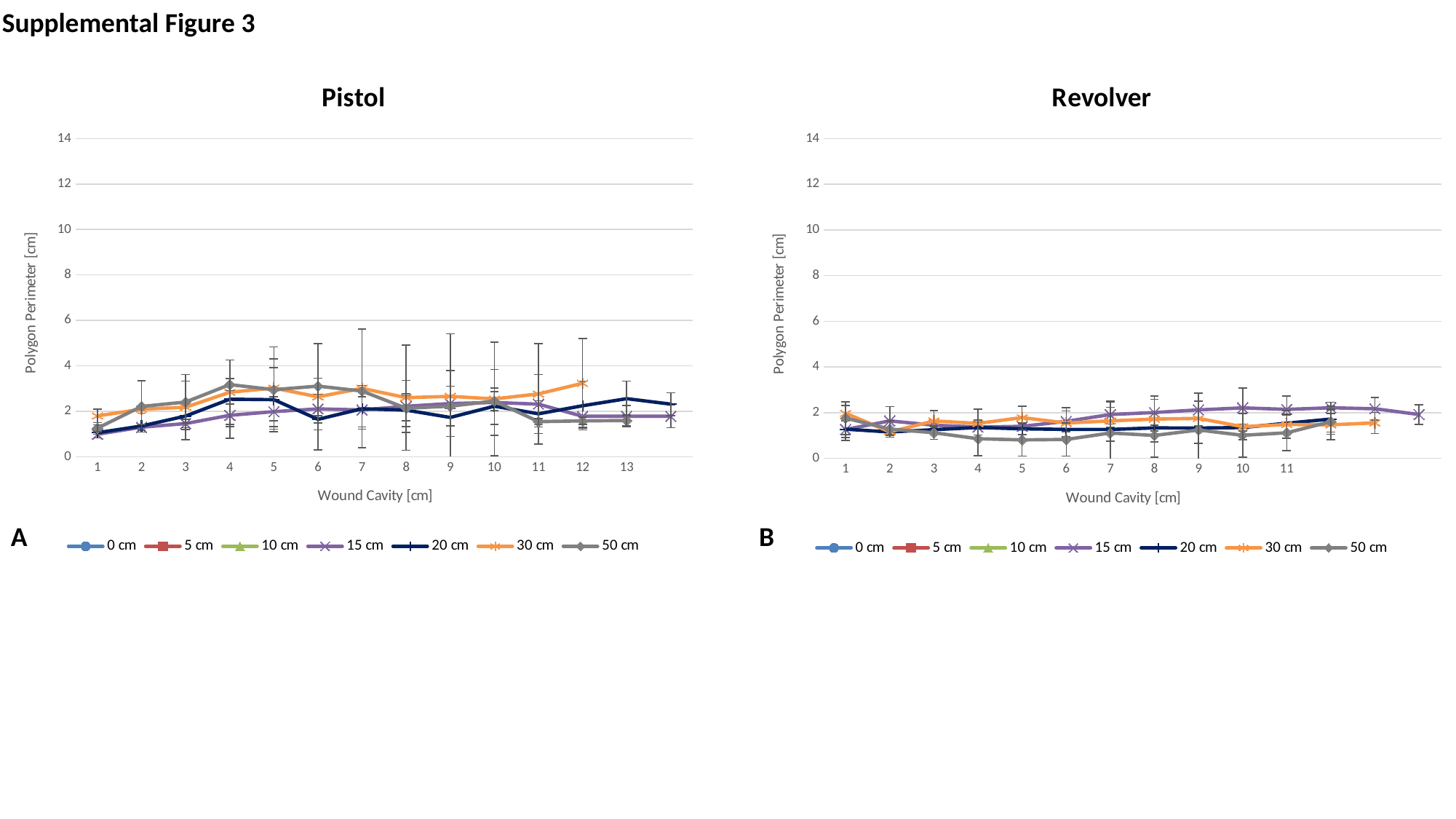

Supplemental Figure 3
### Chart: Pistol
| Category | 0 cm | 5 cm | 10 cm | 15 cm | 20 cm | 30 cm | 50 cm |
|---|---|---|---|---|---|---|---|
### Chart: Revolver
| Category | 0 cm | 5 cm | 10 cm | 15 cm | 20 cm | 30 cm | 50 cm |
|---|---|---|---|---|---|---|---|A
B
